# Association between carotid atherosclerosis and brain activation patterns during the Stroop task in older adults: An fNIRS investigation

**DOI:** 10.1016/j.neuroimage.2022.119302

**Published:** 2022-08-15

**Authors:** Sarah A. Mason, Lamia Al Saikhan, Siana Jones, Sarah-Naomi James, Heidi Murray-Smith, Alicja Rapala, Suzanne Williams, Carole Sudre, Brian Wong, Marcus Richards, Nick C. Fox, Rebecca Hardy, Jonathan M. Schott, Nish Chaturvedi, Alun D. Hughes

**Affiliations:** a MRC Unit for Lifelong Health and Ageing at University College London, Department of Population Science and Experimental Medicine, Institute of Cardiovascular Science, 1–19 Torrington Place, London, WC1E 7HB, United Kingdom; b Department of Cardiac Technology, College of Applied Medical Sciences, Imam Abdulrahman Bin Faisal University, 2835 King Faisal Street, Damman, Kingdom of Saudi Arabia; c Dementia Research Centre, Institute of Neurology, University College London, London, UK; d Centre for Medical Image Computing, Department of Computer Science, University College London, London UK; e School of Biomedical Engineering, King’s College, London UK

**Keywords:** Functional near infrared spectroscopy (fNIRS), Neurovascular coupling, Stroop task, Cognitive function

## Abstract

There is an increasing body of evidence suggesting that vascular disease could contribute to cognitive decline and overt dementia. Of particular interest is atherosclerosis, as it is not only associated with dementia, but could be a potential mechanism through which cardiovascular disease directly impacts brain health. In this work, we evaluated the differences in functional near infrared spectroscopy (fNIRS)-based measures of brain activation, task performance, and the change in central hemodynamics (mean arterial pressure (MAP) and heart rate (HR)) during a Stroop color-word task in individuals with atherosclerosis, defined as bilateral carotid plaques (n = 33) and healthy age-matched controls (n = 33). In the healthy control group, the left prefrontal cortex (LPFC) was the only region showing evidence of activation when comparing the incongruous with the nominal Stroop test. A smaller extent of brain activation was observed in the Plaque group compared with the healthy controls (1) globally, as measured by oxygenated hemoglobin (p = 0.036) and (2) in the LPFC (p = 0.02) and left sensorimotor cortices (LMC)(p = 0.008) as measured by deoxygenated hemoglobin. There were no significant differences in HR, MAP, or task performance (both in terms of the time required to complete the task and number of errors made) between Plaque and control groups. These results suggest that carotid atherosclerosis is associated with altered functional brain activation patterns despite no evidence of impaired performance of the Stroop task or central hemodynamic changes.

## Highlights

• Cognitively normal individuals aged 72 - 73 recruited from a UK-based National Birth Cohort with (n = 33) and without carotid atherosclerosis (n = 33) underwent a Stroop color word task with concurrent assessment of functional near infrared spectroscopy (fNIRS) and hemodynamics.
• Carotid atherosclerosis was associated with a decrease in the extent of brain activation as measured by fNIRS in response to the Stroop test.
• The differences were observed despite no differences in time required to complete the task or number of errors made, or changes in mean arterial pressure or heart rate.
• The results suggest that carotid atherosclerosis is associated with alterations in functional brain activation patterns without impaired Stroop task performance.

## 1 Introduction

A variety of cardiovascular risk factors or cardiovascular disease biomarkers are associated with impaired cognitive function. For example, carotid stenosis (Mathiesen et al., 2003), increased arterial stiffness (Kearney-Schwartz et al., 2009), poorer cardiac function (Sabayan et al., 2015), and carotid atherosclerosis (defined as a thickening and hardening of arteries due to a buildup of plaque in the lumen) (Romero et al., 2009) have been associated with poor performance in tests of attention, memory, processing speed, and executive function. Furthermore, there is evidence suggesting that cardiovascular risk factors are not only associated with cognitive impairment and Alzheimer’s disease (AD), but may in fact precede and/or accelerate the onset of neurodegeneration (Bell, Zlokovic, 2009, Breteler, 2000, De La Torre, 2012, Honjo, Black, Verhoeff, 2012, Kovacic, Castellano, Fuster, 2012). It has been posited that vascular dysfunction or damage arising from cardiovascular risk factors can lead to chronic hypoperfusion and ischemia, impaired cerebral blood flow regulation, and disruption to the blood-brain barrier (Bell and Zlokovic, 2009). According to the Vascular Hypothesis (de la Torre, 2018, de la Torre, Mussivand, 1993), these hemodynamic disturbances not only cause direct brain injury, but can initiate cerebral angiopathy by triggering over-production and reduced clearance of amyloid β-protein, thereby leading to deficits in cognitive domains such as executive function and memory, and potentially resulting in overt dementia (Bell, Zlokovic, 2009, De La Torre, 2012, Honjo, Black, Verhoeff, 2012, Rensink, De Waal, Kremer, Verbeek, 2003).

Atherosclerosis underlies the majority of cardiovascular diseases and is relevant to cognition given that it is not only associated with dementia (Hofman et al., 1997) and an increase in cerebral amyloid deposits (Honig, Kukull, Mayeux, 2005, Li, Cao, Garber, Kim, Fukuchi, 2003), but could be a potential mechanism through which cardiovascular disease directly impacts brain health (Honjo et al., 2012). To better understand the potential role of atherosclerosis in cognitive impairment, we sought to compare (1) cognitive performance, (2) central hemodynamic changes, and (3) patterns of functional brain activity using functional near infrared spectroscopy (fNIRS) in older adults with and without carotid atherosclerosis during an incongruous Stroop color-word task.

The participants in this work were part of the Insight 46 neuroscience sub-study of the National Survey of Health and Development (NSHD). NSHD is an ongoing British birth cohort study where social, lifestyle, cognitive and physical data from over 5000 individuals born within the same week in 1946 has been assessed over the course of the participants’ lives (James, Lane, Parker, Lu, Collins, Murray-Smith, Byford, Wong, Keshavan, Buchanan, Keuss, Kuh, Fox, Schott, Richards, 2018, Kuh, Pierce, Adams, Deanfield, Ekelund, Friberg, Ghosh, Harwood, Hughes, Macfarlane, Mishra, Pellerin, Wong, Stephen, Richards, Hardy, 2011, Kuh, Wong, Shah, Moore, Popham, Curran, Davis, Sharma, Richards, Stafford, Hardy, Cooper, 2016). A sub-sample of 502 NSHD participants volunteered for the Insight 46 study, which is designed to explore preclinical dementia and the causes/consequences of cerebrovascular amyloid pathology on brain health through a battery of clinical, neurosychological, imaging, biomarker, genetic, and vascular assessments (Lane, Parker, Cash, Macpherson, Donnachie, Murray-Smith, Barnes, Barker, Beasley, Bras, Brown, Burgos, Byford, Jorge Cardoso, Carvalho, Collins, De Vita, Dickson, Epie, Espak, Henley, Hoskote, Hutel, Klimova, Malone, Markiewicz, Melbourne, Modat, Schrag, Shah, Sharma, Sudre, Thomas, Wong, Zhang, Hardy, Zetterberg, Ourselin, Crutch, Kuh, Richards, Fox, Schott, 2017, Mason, Al Saikhan, Jones, Bale, James, Murray-Smith, Rapala, Williams, Wong, Richards, Fox, Hardy, Schott, Chaturvedi, Hughes, 2020). Insight 46 provides a powerful opportunity to investigate the mechanisms of early cognitive decline as many participants in this study (aged 72 - 73) likely exhibit subclinical pathophysiological changes such as amyloid pathology and atherosclerosis yet have a low risk of overt dementia (Prince et al., 2014).

The extensive phenotyping protocol performed as part of the Insight 46 study involves psychometric testing using fNIRS (Mason et al., 2020). fNIRS is an optical technique whereby changes in concentration of oxygenated hemoglobin (O2Hb) and deoxygenated hemoglobin (HHB) in the cerebral microvasculature of the cortical surface can be detected (Bunce, Izzetoglu, Izzetoglu, Onaral, Pourrezaei, 2006, Scholkmann, Kleiser, Metz, Zimmermann, Mata Pavia, Wolf, Wolf, 2014). As fNIRS is non-invasive and relatively insensitive to motion, it can be used to evaluate hemodynamic changes arising from neurovascular coupling (NVC) during a variety of cognitive tasks in a naturalistic environment (Pinti, Merla, Aichelburg, Lind, Power, Swingler, Hamilton, Gilbert, Burgess, Tachtsidis, 2017, Udina, Avtzi, Durduran, Holtzer, Rosso, Castellano-Tejedor, Perez, Soto-Bagaria, Inzitari, 2020). NVC refers to the localised increase in blood flow that occurs in response to a cognitive stimulus, and is commonly used as a surrogate for neuronal activity.

In this work, we assessed fNIRS-based measures of brain activity in older adults during the Stroop color-word task, which is a well-established method for evaluating executive function, specifically in the ability to inhibit automatic responses (Scarpina, Tagini, 2017, Stroop, 1935, Vendrell, Junqué, Pujol, Jurado, Molet, Grafman, 1995). The degree of Stroop interference (both in terms of response time and the number of errors made) not only increases as a function of normal ageing (Davidson et al., 2003), but is exacerbated by conditions such as cognitive impairment and dementia (Amieva, Lafont, Rouch-Leroyer, Rainville, Dartigues, Orgogozo, Fabrigoule, 2004, Laguë-Beauvais, Brunet, Gagnon, Lesage, Bherer, 2013, Spieler, Balota, Faust, 1996).

Here, we use the presence of ultrasound-diagnosed bilateral carotid plaques as an indicator of moderately severe atherosclerotic burden, and assess whether there are any differences in performance, hemodynamics, and/or brain activation patterns in atherosclerotic individuals compared with age-matched controls. We hypothesize that atherosclerotic individuals will perform significantly worse than the healthy controls on the Stroop task in terms of both time to complete the task and the number of errors made. NVC is identified in regions where there is a concurrent increase in O2Hb concentration and decrease in HHB concentration. We aim to compare fNIRS-measures of changes in O2Hb and HHB between groups between corresponding anatomical brain regions to determine the strength and location of NVC. We hypothesize that the presence of bilateral carotid plaques will be associated with a significant reduction in NVC primarily in the prefrontal cortices when comparing the incongruous with the nominal Stroop test.

## 2 Methods

### 2.1 Participant recruitment

Participants were selected from 184 participants (aged 72 - 73) who attended an Insight 46 clinic between October 2018 - March 2020. Research Ethics Committees in England and Scotland provided approval for NSHD (Hardy, Ghosh, Deanfield, Kuh, Hughes, 2016, Kuh, Pierce, Adams, Deanfield, Ekelund, Friberg, Ghosh, Harwood, Hughes, Macfarlane, Mishra, Pellerin, Wong, Stephen, Richards, Hardy, 2011, Kuh, Wong, Shah, Moore, Popham, Curran, Davis, Sharma, Richards, Stafford, Hardy, Cooper, 2016, Lane, Parker, Cash, Macpherson, Donnachie, Murray-Smith, Barnes, Barker, Beasley, Bras, Brown, Burgos, Byford, Jorge Cardoso, Carvalho, Collins, De Vita, Dickson, Epie, Espak, Henley, Hoskote, Hutel, Klimova, Malone, Markiewicz, Melbourne, Modat, Schrag, Shah, Sharma, Sudre, Thomas, Wong, Zhang, Hardy, Zetterberg, Ourselin, Crutch, Kuh, Richards, Fox, Schott, 2017). The National Research Ethics Service Committee London (REC reference 14/LO/1173) approved the Insight 46 sub-study. All participants provided written informed consent to participate and for their data to be stored. Exclusion criteria for the study included: a Mini-Mental State Exam (MMSE) below 24, established cardiovascular disease at age 53, heart attack by the age of 69.

### 2.2 Investigations

The Insight 46 protocols have been previously described (Mason et al., 2020). All participants completed a health and lifestyle questionnaire and underwent measurements of height and weight, blood pressure, and blood tests. Cholesterol/high density lipoprotein (HDL) ratio was calculated for each participant from the most recently available data from the NSHD database. The MMSE score from each participant was also obtained from the most recently available data from the NHSD database to confirm that participants had normal cognitive function. An EPIQ 7G ultrasound scanner (Philips Healthcare, Andover MA, USA) with a linear array transducer (L12-3) was used to check for the presence of plaques in the left and right carotid arteries. A plaque was defined as a focal structure that encroaches into the arterial lumen by at least 0.5 mm or 50% of the surrounding intima-media thickness or a region of intima-media thickness >1.5 mm (Touboul et al., 2012). Echocardiography was performed using an EPIQ 7G ultrasound scanner with an X5-1 transducer. A 3-lead electrocardiogram (ECG) was recorded concurrently with all ultrasound examinations. In addition, a standard 12-lead resting ECG was performed in the supine position. All abnormal findings (presence of carotid plaques, dilated atria, atrial fibrillation, left bundle branch block, aortic valve calcification, left ventricular hypertrophy, low voltage QRS etc.) were recorded in the electronic case report form.

Findings on vascular ultrasound, echocardiography, and 12-lead electrocardiography (ECG) were used to categorize the study participants based on their cardiovascular health at the time of their second Insight 46 clinic visit. Participants were tagged with at least one label as shown in Table 1 depending on the number and type of incidental findings from the cardiovascular assessment. Participants with label 0 (no incidental findings) were classified as ‘Healthy’ and participants with label 3 (more severe atherosclerosis, with plaque detected on both the left and right carotid artery) were categorized into the ‘Plaque’ group. Note that participants with unilateral plaques were excluded from further analysis.

**Table 1:** Labels used for participant categorization based on incidental findings from the cardiovascular assessment.

Label	Definition
0	No incidental findings
1	Carotid plaque (left side only)
2	Carotid plaque (right side only)
3	Carotid plaques (both sides)
4	Abnormal ECG
5	Other

### 2.3 Stroop color-word testing protocol

A verbal Stroop color-word task was performed. Though there are different versions of the Stroop test, the basic concept is to measure the accuracy and speed with which one can identify the color of a word printed in ink mismatched to the meaning of the color (i.e. the word ‘blue’ printed in green ink). As language is a powerful distractor, there is a natural delay in response time in this task compared with identifying the color of non-words (such as ‘XXXXX’ printed in green ink, for example). Our implementation of the Stroop task consisted of three blocks, with each block preceded and followed by a 60-second baseline/recovery period where the participant stared straight ahead at a black poster. All blocks were presented on white A4 laminated sheets of paper held upright by a free-standing clipboard to avoid a downward head-tilt whilst reading. The description of each block of the Stroop task is described below:

• Block 1 - **Nominal**: Participants read aloud 100 words (spelling either ‘blue’, ‘red’, or ‘green’) printed in black ink in five evenly spaced columns on the page
• Block 2 - **Congruous**: Participants read aloud colors of 100 ‘XXXXX’ (printed in either blue, red, or green ink) displayed in five evenly spaced columns on the page
• Block 3 - **Incongruous**: Participants read aloud the color (red, blue or green ink) of 100 words (spelling ‘red’, ‘green’, or ‘blue’), where the color of the word was mismatched to the meaning of the word itself. The words were displayed in five evenly spaced columns on the page.

The number of errors made and time required to complete each Stroop block were recorded to quantify task performance.

### 2.4 Hemodynamic and fNIRS monitoring during the stroop task

**Fig. 1:**
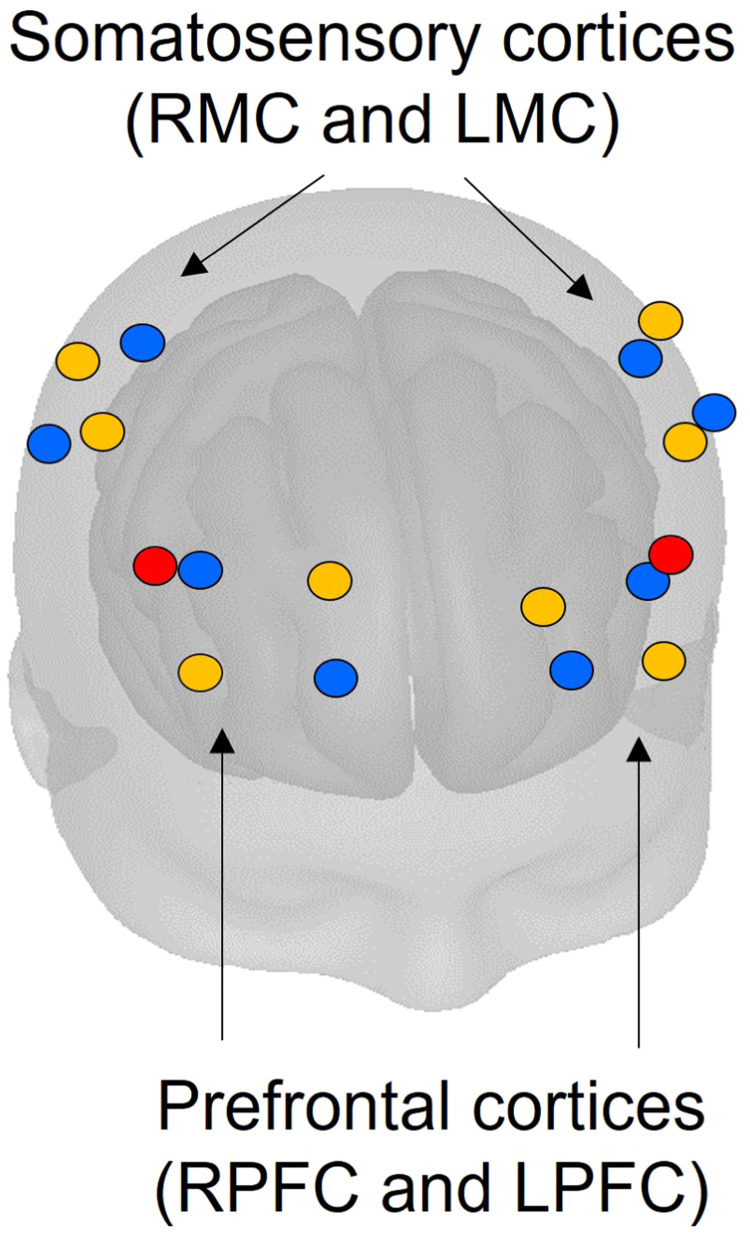
An example of the channel position of the Brite 24 cap obtained with the Pohemus digitization. Sources are shown in orange (long separation channels) and red (short separation channels). Detectors are shown in blue. Note that the sources and detectors are arranged in quadrants over the left and right somatosensory cortices and prefrontal cortices.

Throughout the duration of the Stroop task, cerebral hemodynamics were monitored with an 18-channel, dual-wavelength (760 and 850 nm) continuous wave Brite 24 fNIRS device (Artinis Medical Systems, BV, Zetten, The Netherlands). This device had 16 long separation channels (where the source and detector were separated by 3 cm) arranged in quadrants approximately over the left and right prefrontal cortex (LPFC/RPFC) and the left and right motor/somatosensory cortices (LMC/RMC). This device also had 2 short separation channels (where the source and detector were separated by 1 cm) placed on the left and right PFC. See Fig. 1 for an illustration of channel positioning. The sampling frequency of this device was 10 Hz. The investigator positioned the cap symmetrically on the participant’s head with the brim approximately 1 cm above the eyebrows, and secured the position of the cap using the chin strap. A Polhemus Patriot Device (Polhemus, Colchester, VT, USA) was used to determine the position of each source and detector with respect to anatomical landmarks of the head based on the international 10/20 system (Jurcak et al., 2007). The operator tagged the position of the nasion, inion, left preauricular point, right preauricular point, top of the head along the mid-sagittal plane (Cz), each source, and each detector using the electromagnetic Polhemus sensor. These positions were converted from real space to the Montreal Neurological Institute (MNI) stereotaxic coordinate system automatically in the software used to interface with the Brite 24 (Oxysoft, Artinis Medical Systems, BV, Zetten, the Netherlands).

Changes in beat-to-beat peripheral mean arterial pressure (MAP) and heart rate (HR) were also monitored during the Stroop task using a Finometer (Finapres Medical Systems BV, the Netherlands) placed on the finger cuff on either the index finger or middle finger of the non-dominant hand according to the manufacturer’s instructions. Finometer readings were synchronized with fNIRS readings using electronic markers to indicate the start and end of each Stroop and baseline/recovery block.

### 2.5 fNIRS signal processing pipeline

All analyses were performed in Matlab® (2021a) using the SPM-fNIRS toolbox developed by the NeuroImaging Tools & Resources Collaboratory (NITRC) (Tak et al., 2016). This software enabled statistical parametric mapping of fNIRS data using SPM12 software.

#### 2.5.1 Temporal preprocessing and channel rejection

**Fig. 2:**
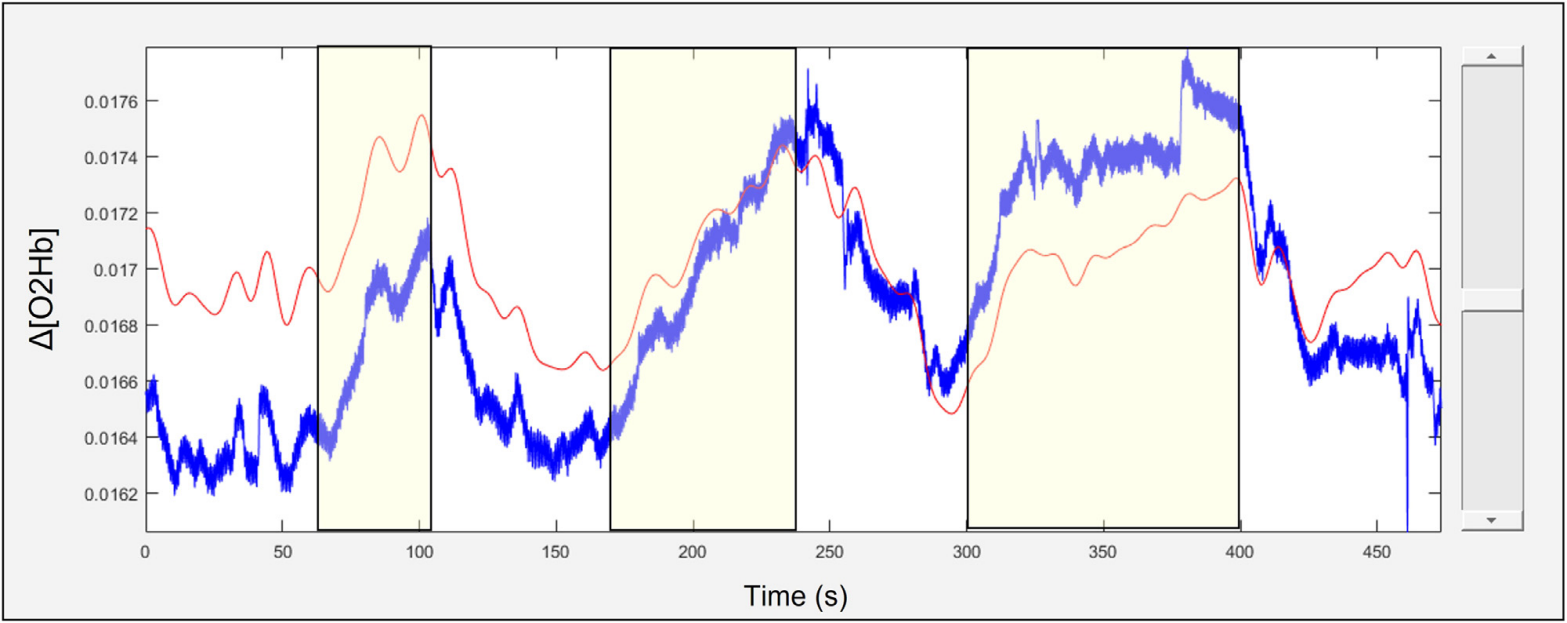
An example of unprocessed (blue) and fully processed (red) fNIRS data obtained from a participant performing the verbal Stroop task. The trace shown is ΔO2Hb in units of mol/L. The stimulus periods (Stroop blocks 1, 2, and 3) are indicated by the yellow rectangles superimposed over the fNIRS traces.

fNIRS Intensity data from each channel was converted into ΔO2Hb and ΔHHB using the Modified Beer Lambert Law and differential pathlength factors (DPF) of 7.4 and 6.4 for the 760 nm and 850 nm wavelengths respectively. These DPFs were calculated automatically in the SPM-fNIRS software using the age-dependent general equation for the DPF derived by Scholkmann and Wolf (Scholkmann and Wolf, 2013). Motion artefacts were corrected using the Motion Artifact Reduction Algorithm (MARA) with the default values of 1, 3, and 5 accepted for the moving window length (L), threshold factor-motion detection (th) and smoothing factor motion artifact (α) parameters respectively (Scholkmann et al., 2010). A band-stop filter with cut-off frequencies of 0.1 and 2 Hz was applied to remove physiological noise. The data were then downsampled to 1 Hz and linear de-trended using a discrete cosine transform (DCT) with a cutoff of 160 seconds. An example channel from a study participant before and after temporal preprocessing is shown in Fig. 2.

The frequency spectrum of the ΔO2Hb for each channel (before filtering) was visually assessed in the SPM-fNIRS temporal preprocessing GUI to check for a peak at around 1 Hz corresponding to the cerebrovascular pulsatility arising from the heartbeat. This is a well-established indicator of fNIRS signal quality as it demonstrates good optical coupling has been achieved (Yücel et al., 2021). Any trace with a very small or non-existent peak was visually inspected further in the time domain to confirm poor data quality. Any trace with poor optical coupling (arising from either an over-saturation of ambient light or poor contact with the participant’s scalp) or completely corrupted by motion artefacts was rejected and not analysed further. Each participant was given an overall fNIRS signal quality rating based on the following criteria:

1. **Unacceptable**: Over half of the channels were rejected, and/or 02Hb and HHB were suspiciously correlated due to motion artefacts not adequately corrected for by the MARA algorithm
2. **Acceptable**: At least half of the channels retained, with zero to minor detectable artefacts

Only participants with a rating of ‘Acceptable’ were considered for further analysis.

#### 2.5.2 Model specification and estimation

A general linear model (GLM) was used to analyse the temporally preprocessed O2Hb and HHB fNIRS signals. The GLM is shown in Eq. 1 (Huppert, 2016, Pinti, Merla, Aichelburg, Lind, Power, Swingler, Hamilton, Gilbert, Burgess, Tachtsidis, 2017, Scholkmann, Kleiser, Metz, Zimmermann, Mata Pavia, Wolf, Wolf, 2014), where Y is the measured fNIRS signal after processing, X is the design matrix, β is the vector of weights of each regressor in the design matrix, and ϵ is the error. The Model Specification routine in SPM-fNIRS was used to (1) correct for serial autocorrelations in the fNIRS data using a first order autoregressive (AR(1)) plus white noise model (Friston, Glaser, Henson, Kiebel, Phillips, Ashburner, 2002, Purdon, Weisskoff, 1998) and (2) construct the design matrix, which consisted of:

1. A task regressor for each of the three Stroop blocks modelled using the canonical hemodynamic response function (HRF) convolved with the box car function corresponding to individual task duration with time and dispersion derivatives to allow for the peak and width of the response to vary by plus or minus a second, respectively (Martin A. Lindquist et al., 2009).
2. The left and right short separation channels (after preprocessing as described in Section 2.5) to remove extracerebral contamination (Scholkmann, Kleiser, Metz, Zimmermann, Mata Pavia, Wolf, Wolf, 2014, Yücel, Selb, Aasted, Petkov, Becerra, Borsook, Boas, 2015).
3. A constant to model the signal mean.

(1) Y=X·β+ϵ

The β coefficients were solved for on a channel by channel basis using the Model Estimation routine in SPM-fNIRS.

### 2.6 Evaulation of hemodynamic effects

Equation 2 indicates how channel-wise T statistics were calculated given a contrast vector c and the covariance of the β coefficients (see Eq. 3) calculated using the GLMHuppert (2016). The nominal Stroop task was chosen as our control as the experimental conditions were identical to the incongruous Stroop task (i.e. reading and speaking aloud), but the PFC was not activated as this task is considered an ‘automatic’ process. We therefore assessed whether there was significantly greater evidence of NVC in the incongrous Stroop task (Block 3) compared to the nominal Stroop task (Block 1) by setting the contrast vector values to 1 and -1 for the Block 3 task regressor and Block 1 task regressor, respectively.

(2) $$T=c\cdot \beta /\sqrt{c\cdot Cov_{b}\cdot c^{T}}$$

(3) $$Cov_{\beta }={\left(X^{T}\cdot X\right)}^{-1}\cdot \sigma^{2}$$

### 2.7 Statistical analyses

#### 2.7.1 Stroop performance

The two performance metrics considered were: (1) time required to complete the incongruous Stroop task and (2) the number of errors made. Data were reported as medians and nonparametric 95% CIs. Differences in both of these performance metrics between the Healthy and Plaque group were determined using a Mann-Whitney *U* test.

#### 2.7.2 Central hemodynamics during Stroop task

Data were reported as means ± SD. Student’s t-tests were used to test for differences between the Healthy and Plaque groups in the change in MAP and HR during the incongruous Stroop task relative to the preceding baseline period.

#### 2.7.3 Cerebral fNIRS during Stroop task

**Fig. 3:**
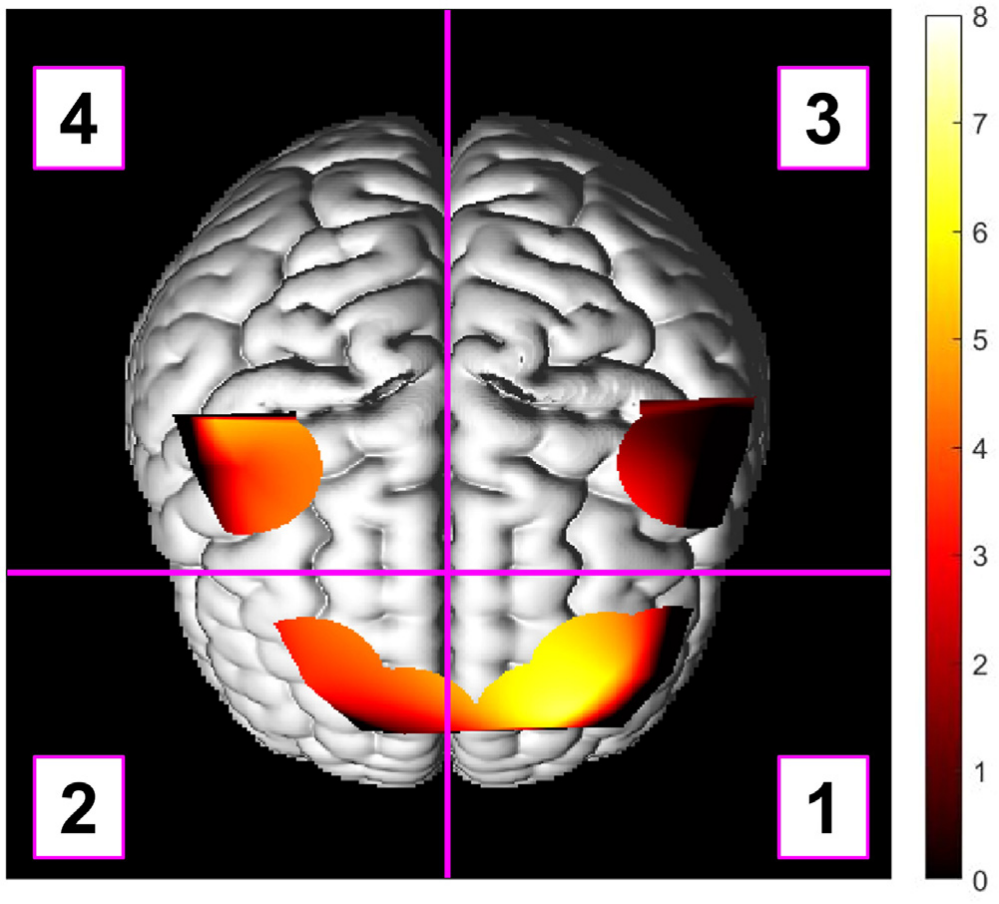
An example of how the t statistic map superimposed over a rendered brain was subdivided into the four anatomical regions used in the statistical analysis. Region 1 = left prefontal cortex (LPFC), Region 2 = right prefrontal cortex (RPFC), Region 3 = left sensorimotor cortices (LMC), and Region 4 = right sensorimotor cortices(RMC).

The t statistic maps generated for each participant for O2Hb and HHB were subdivided into four anatomical regions as shown in Fig. 3: (1) the left prefrontal cortex (LPFC), (2) the right prefrontal cortex (RPFC), (3) the left sensorimotor cortices (LMC), and (4) the right sensorimotor cortices (RMC). In addition to comparing the overall activation between the Healthy and Plaque groups in the incongruous relative to the nominal condition, we postulated a priori that there would be effect modification by anatomical region as we expect minimal activation in the sensorimotor regions, high activation in the prefrontal regions, and a potential hemispheric effect depending on the dominant hand of the participant. Therefore, the mean T statistic for each region in the incongruous relative to the nominal condition was computed for each participant and was used as the outcome variable in a robust linear mixed effects (LME) model with anatomical region and group (Healthy vs. Plaque) as fixed effects and participant ID as a random effect for O2Hb and HHB separately. The LME analysis was performed using Stata 17 (StataCorp. 2021. Stata Statistical Software: Release 17. College Station, TX: StataCorp LLC.), other statistical analyses were performed using Matlab 2021a. The RMC was used as the contrast region in the LME model, as we expect this region to have the lowest level of NVC during the Stroop task because (a) the Stroop task is not designed to activate motor functions and (b) the right hemisphere may be less dominant given the handedness of the majority of the participants. The Huber White Sandwich estimator was used to improve estimates of standard error given the heteroskedasticity of the data. Statistical significance was assigned using the conventional threshold of p < 0.05.

## 3 Results

### 3.1 Participant characteristics

102 out of 184 fNIRS datasets received a rating of 2 (acceptable quality), and were therefore considered for further analysis. Of these 102 participants, 33 participants had no incidental findings and were included in the Healthy group and 33 participants had plaque on both the left and right carotid arteries and were included in the Plaque group. Note, the groups were not planned to be matched 1:1, and it is coincidental that there were 33 participants exactly in each group. Table 2 summarizes the sex, handedness, and other cardiovascular health phenotypes assessed for the participants within each group. There were significantly more men in the Plaque group (p=.007), but otherwise the two groups were similar.

**Table 2:** Participant characteristics for each group.

	Healthy (n = 33)	Plaque (n = 33)
**Age** (**mean**±**SD**)	73.7 ± 0.7	73.6 ±0.5
**Men**	12 (36%)	23 (70%)
**Right-handed**	31 (94%)	30 (91%)
**Cardiovascular disease at age 53**	0 (0%)	0 (0%)
**Type 2 diabetes at age 63**	2 (6%)	3 (9%)
**Smoking status** : Current smokers	0 (0%)	2 (6%)
Ex-smokers	15 (45%)	21 (64%)
Non-smokers	18 (55%)	9 (27%)
Question not answered	0 (0%)	1 (3%)
**BMI (age 69 - 71, mean**±**SD)**	27.8 ± 3.5	27.9 ± 4.2
**Systolic blood pressure (age 69 - 71, mean**±**SD)**	133.4 ± 15.2	137.1 ± 14.6
**Diastolic blood pressure (age 69 - 71, mean**±**SD)**	74.0 ± 8.1	75.0 ± 8.9
**Cholesterol/HDL ratio (age 69 - 71, mean**±**SD)**	3.4 ±- 1.1	3.3 ± 1.2
**MMSE score (age 69 - 71, mean**±**SD)**	29.4 ± 1.0	29.5 ± 1.0

### 3.2 Performance in Stroop task

The median [95% CI] time to complete the incongruous Stoop task was 104s [98, 122] and 109s [97, 133] for the Healthy and Plaque groups respectively. The median [95% CI] number of errors made during the incongruous Stroop task was 1 [1, 3] and 2 [1, 2] for the Healthy and Plaque groups respectively. There was no evidence of a difference between the Healthy and Plaque groups in either the time required to complete the task or the number of errors made (p = 0.43 and 0.99, respectively). Both the Healthy and Plaque groups made zero errors in the nominal condition of the Stroop Task.

### 3.3 Central hemodynamics during Stroop task

There was no evidence of differences in the change in HR or MAP associated with the incongruous Stroop test between groups with respect to (1) the baseline period or (2) the nominal Stroop task.

(1) The mean ± SD change in MAP with respect to the baseline period was 5.8 ± 5.1 mmHg and 7.3 ± 4.1 mmHg for the Healthy and Plaque groups respectively (p = 0.24). The mean ± SD change in HR with respect to the baseline period was 5.0 ± 3.5 beats/min and 3.7 ± 2.4 beats/min for the Healthy and Plaque groups respectively (p = 0.13).

(2) The mean ± SD change in MAP with respect to the nominal Stroop test was 5.04 ± 6.4 mmHg and 6.8 ± 7.0 mmHg for the Healthy and Plaque groups respectively (p = 0.38). The mean ± SD change in HR with respect to the nominal Stroop test was 0.7 ± 3.9 beats/min and 0.67 ± 1.7 beats/min for the Healthy and Plaque groups respectively (p = 0.93).

### 3.4 Cerebral fNIRS during Stroop task

Out of the 1188 channels available for analysis (18 channels times 66 participants), 41 were rejected in total. The average number of channels excluded per participant was 0.61.

**Fig. 4:**
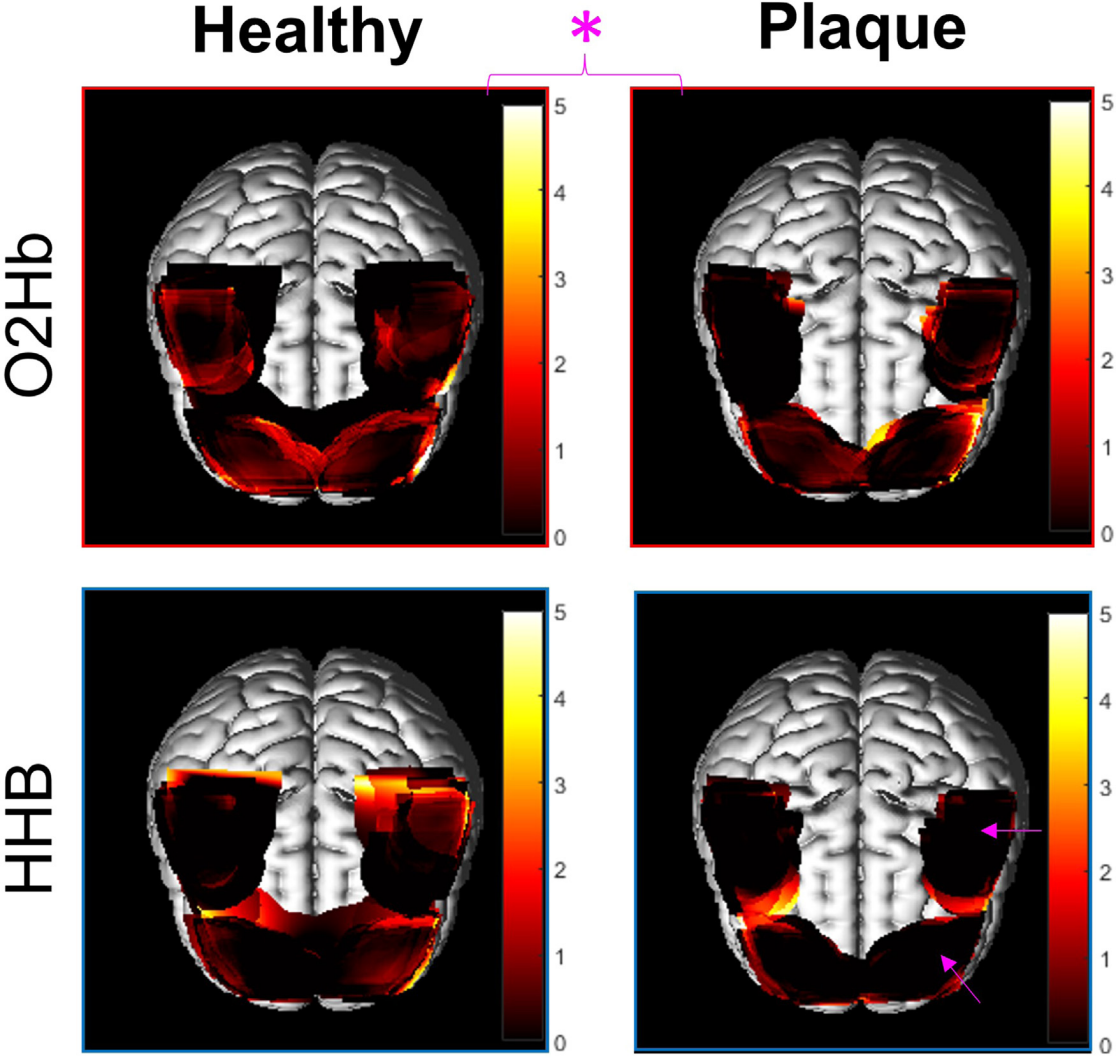
Shows the mean t statistic maps for each group (Healthy and Plaque) when comparing the incongruous Stroop to the nominal Stroop for oxygenated haemoglobin (O2Hb) and deoxygenated haemoglobin (HHB). The higher the t statistic, the greater evidence of increased change in Hb (rise in O2Hb, fall in HHB), and thus functional brain activity. Note that the average t statistic is significantly lower overall in the Plaque group compared with the Healthy group in O2Hb as indicated by the pink asterisk. Also note the significant reduction in t statistic in the LPFC and LMC in the Plaque group compared with the Healthy group in HHB as indicated by the pink arrows.

Figure 4 shows the mean t statistic maps for the Healthy and Plaque groups for O2Hb and HHB. The direction of expected change in response to the stimulus (positive for O2Hb and negative for HHB) was incorporated into the GLM such that a positive t value corresponds to a greater increase in O2Hb and a greater decrease in HHb with respect to the nominal condition. Note that there is evidence of increased O2Hb in response to the incongruous condition in all brain regions in the Healthy group, and in the RPFC, LPFC, and LMC in the Plaque group (prevalence of t values > 2).

The LME coefficient indicating the effect of being categorized into the Plaque group for O2Hb was -1.13 (p = 0.036) which suggests that plaque is associated with a global reduction in O2Hb for all regions. However, there were no significant differences between individual regions observed in the LME for O2Hb for (i) all participants regardless of group and (ii) when an interaction between group and region was included. The output from the LME analysis is given in the Supplementary Information (S1).

In the LME for HHB, the LPFC of all participants (regardless of group) showed a significantly greater drop in HHB compared to the control region, (coefficient = 0.65, p = 0.042). There were no significant differences between Healthy and Plaque groups (p = 0.77), which indicates that no global reduction in HHB was detected. However, significant reductions in HHB in specific regions were observed when including an interaction between Group and Region. The LPFC (coefficient = -1.36, p = 0.021) and LMC (coefficient = -1.25, p = 0.008) showed significantly a significantly smaller decrease in HHB in the Plaque group compared to the Healthy group as indicated by the magenta arrows in Fig. 4. The output from the LME analysis for HHB is given in Supplementary Information S1.

Together, the results from O2Hb and HHB suggest that NVC (identified by an increase in O2Hb and corresponding decrease in HHB) is significantly reduced in the left hemsiphere of the brain of participants in the Plaque group compared to the Healthy group. Additionally, the HHB LME suggests that general activation in the LPFC is observed for participants in both groups in response to the incongruous Stroop task.

## 4 Discussion

**Fig. 5:**
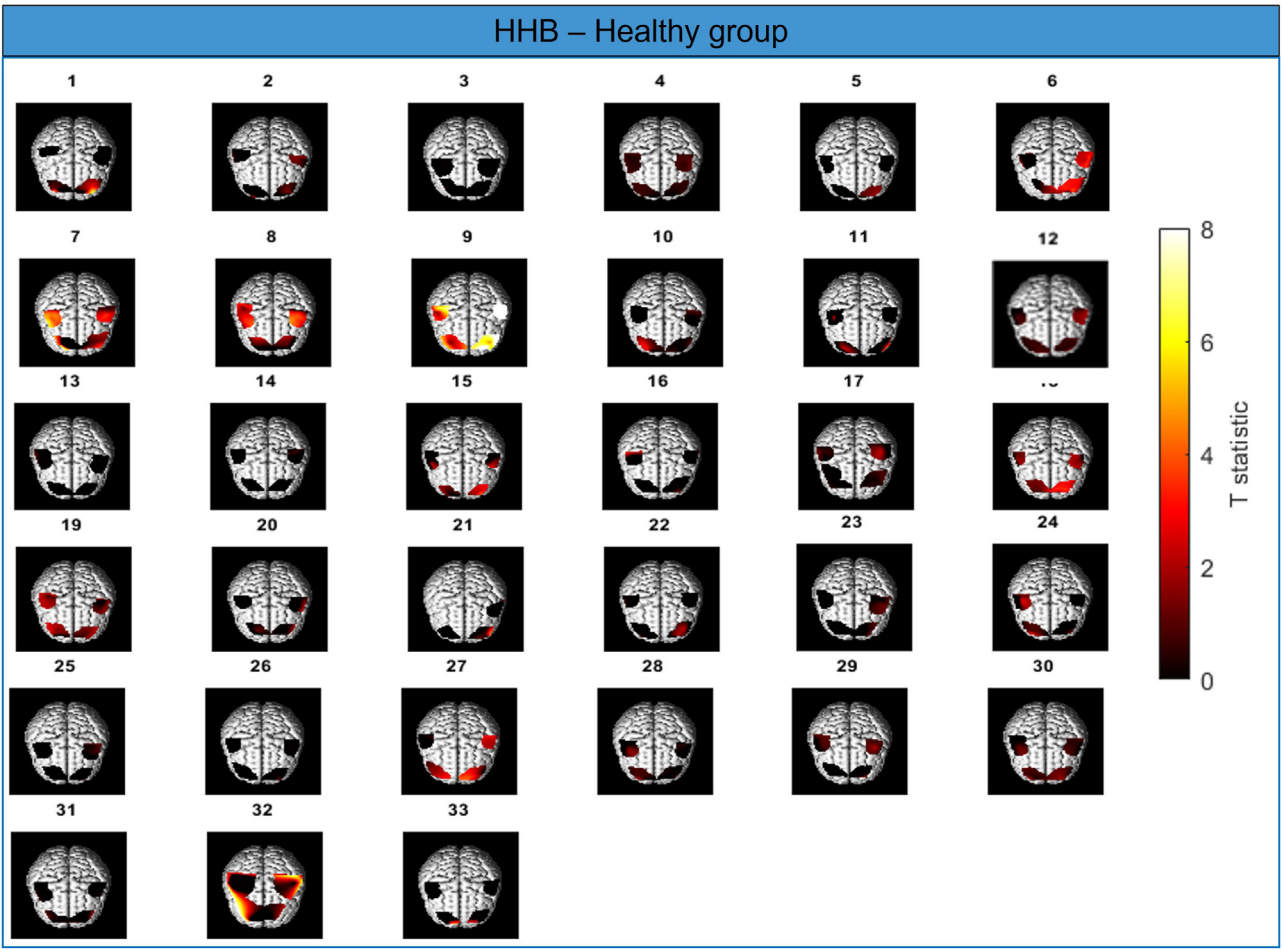
Individual t statistic maps showing the brain activation pattern for all 33 participants in the Healthy group performing the Stroop task (incongruous vs. null condition). The t statistic is indicated by the colorbar, with brighter colors indicating a high t value and thus greater evidence for NVC.

There are various factors contributing to the expected pattern of brain activity during the incongruous Stroop task, such as handedness, age, sex, and brain health (Beratis, Rabavilas, Papadimitriou, Papageorgiou, 2010, Cuevas, Calkins, Susan D. (University of North Carolina, Bell, 2016, Laguë-Beauvais, Brunet, Gagnon, Lesage, Bherer, 2013, Li, Zheng, Wang, Gui, Li, 2009, Milham, Erickson, Banich, Kramer, Webb, Wszalek, Cohen, 2002, Vendrell, Junqué, Pujol, Jurado, Molet, Grafman, 1995). In general, it is widely accepted that the ability to perform a color-word matching Stroop task requires adequate function of a fronto-parietal network including the anterior cingulate cortex (ACC), the PFC, and the parietal lobe (Laird, McMillan, Lancaster, Kochunov, Turkeltaub, Pardo, Fox, 2005, Li, Zheng, Wang, Gui, Li, 2009, Roberts, Hall, 2008, Vanderhasselt, de Raedt, Baeken, 2009). Although the PFC has been shown to be active bilaterally during an incongruous Stroop task (Laguë-Beauvais, Brunet, Gagnon, Lesage, Bherer, 2013, Milham, Erickson, Banich, Kramer, Webb, Wszalek, Cohen, 2002, Zysset, Müller, Lohmann, Von Cramon, 2001), there is some evidence suggesting that the left hemisphere may be more active than the right in young healthy individuals (Jonides et al., 2000). Due to cap geometry and limitations of fNIRS penetration depth, we were unable to assess activity in the ACC and parietal lobe. We observed that on average, all regions for all participants (except for the RMC in the Plaque group) demonstrated an increase in H2Ob in response to the incongruous Stroop task compared with the nominal task as evidenced by the prevalence of relatively high t values (>2) as shown in Fig. 4. Given the global increase in O2Hb, we used HHB (which was more localized) as an arbitrator to determine which regions likely exhibit evidence of NVC, as an increase in H2Ob alone is not sufficient (Pinti et al., 2021). Reassuringly, LME for HHB revealed that all participants had significantly greater decreases in HHB concentration in the LPFC compared to the RMC (p = 0.042). We interpret this to mean that the LPFC has a significantly greater level of NVC than the RMC (i.e. the control region), which is broadly consistent with the expected activation pattern of the PFC based on the literature. However, the HHB LME model did not show significant differences in activation between the RPFC and RMC. This may be due to an age-related loss of localisation of functional activation, resulting in a diffuse, heterogenous, and/or weaker activation in older adults compared with younger adults (Laguë-Beauvais, Brunet, Gagnon, Lesage, Bherer, 2013, Milham, Erickson, Banich, Kramer, Webb, Wszalek, Cohen, 2002, Schroeter, Zysset, Kruggel, Von Cramon, 2003). Indeed, the variability in brain activation patterns between individuals is exemplified in Fig. 5. Although some participants retain the ‘model’ activation pattern characterised by highly focal regions of activation in the bilateral PFC (e.g. participants 1, 11, 33), the majority of participants show a spread in activation (e.g. participants 6, 18, 19, 27) or weak/null global activation (e.g. participants 3, 4, 13, 14, 25, 26) which is expected given the age (72 - 73 years) of the participants in this study. We did not see any convincing evidence of PFC dominance in the O2Hb LME. However, the long stimulus times used made the O2Hb signal particularly susceptible to physiological contamination thereby making it less reliable than the HHB signal Tachtsidis and Scholkmann (2016).

Furthermore, the LME results indicate microvascular cerebral hemodynamics may be altered in individuals with bilateral carotid plaques compared with healthy controls in association with an incongruous Stroop test. Specifically, there appears to by vascular impairment in the Plaque group compared with the Healthy group; this is demonstrated by a significant reduction in the magnitude of HHB decrease in the left hemisphere of the brain, which is accompanied by a significantly reduced increase in O2Hb (though the latter is a global effect).

**Fig. 6:**
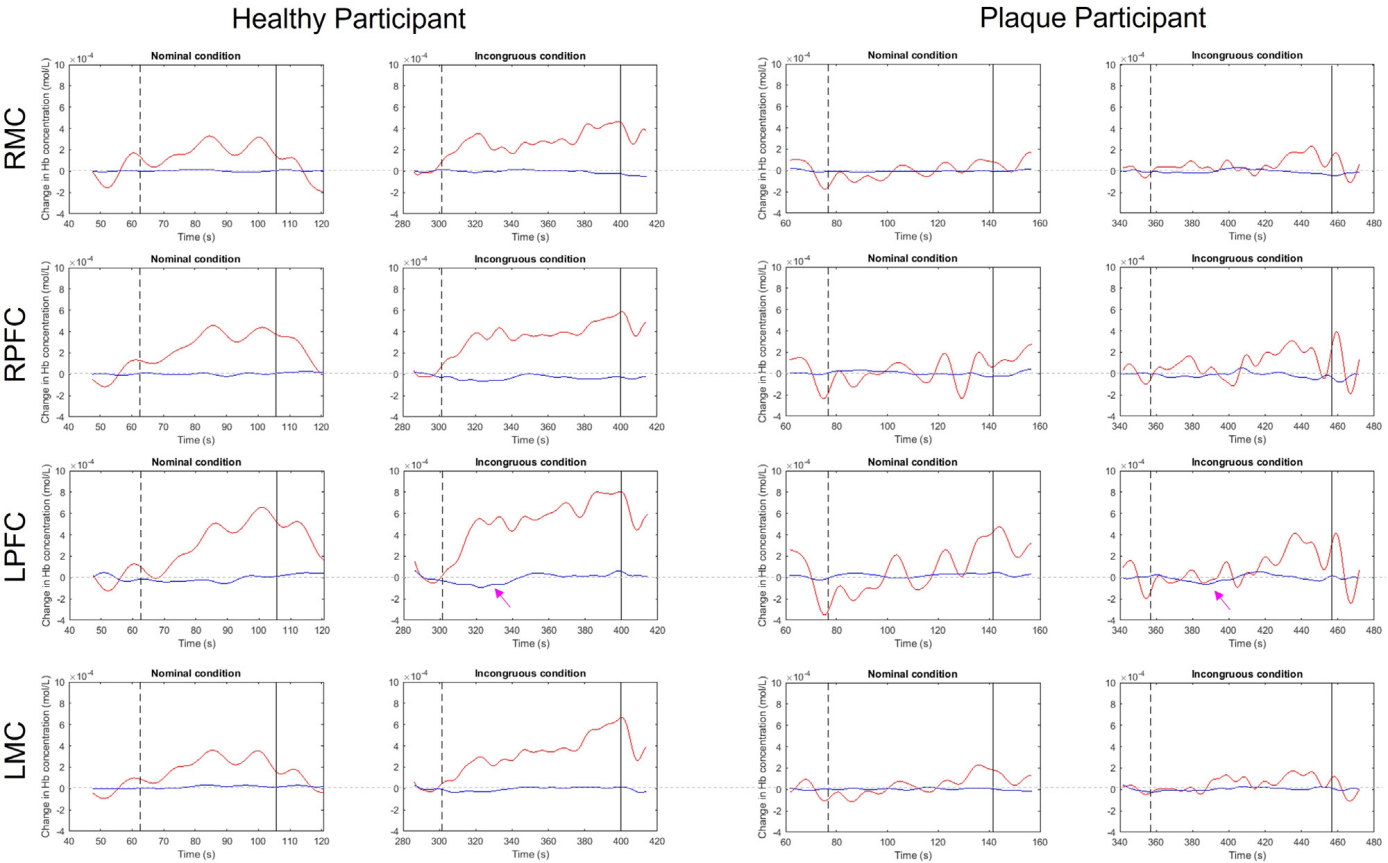
Example channels from each of the four brain regions assessed for an example participant in the (left) Healthy group and the (right) Plaque group. The Hb signals shown here have been temporally pre-processed as described in Section 2.5.1, but otherwise reflect the raw data (i.e. before short separation channel regression with the GLM). Note that overall, the OHB signal (red lines) increases to a greater extent in response to the incongruous condition for the participant in the Healthy group compared to the participant in the Plaque group. Also note the increased drop in HHB (blue lines) in the LPFC in the Healthy participant compared with the Plaque participant (magenta arrows).

Examples of the fNIRS data for individual participants from each group (after temporal pre-processing but before applying the GLM) are shown in Fig. 6. Note the increased magnitude of the OHB response globally for the participant in the Healthy group compared with the participant in the Plaque group. Although smaller in magnitude and not sustained throughout the stimulus, also note that the drop in HHB in the LPFC for the first 40 seconds during the incongruous condition is larger in the Healthy participant compared with the Plaque participant (pink arrows).

The observation that carotid plaques are associated with a reduction in NVC is similar to previous studies investigating the effect of atherosclerosis on cerebral hemodynamics and oxygenation. For example, Khan et. al (2021) used perfusion-weighted MRI to assess the relationship between carotid stenosis and cerebral perfusion and found that over 80% of patients had a detectable reduction in brain perfusion in the hemisphere ipsilateral to the carotid stenosis (Khan et al., 2021). Lal et. al (2017) used the transcranial Doppler (TCD) breath-holding index (BHI) to compare cerebral hemodynamics in patients with asymptomatic carotid stenosis (> 50% occulsion) and age-matched healthy controls, and found a reduced ispsilateral BHI in half of the patients (Lal et al., 2017). Similarly, Vernieri et. al (2006) demonstrated a significant reduction in vasoreactivity using both fNIRS and TCD in patients with symptomatic carotid artery occlusion compared to patients with asymptomatic carotid artery occlusion (Vernieri et al., 2006). Furthermore, Forero et. al (2017) used fNIRS to show that patients with carotid stenosis had (1) a decreased vasoreactivity by 23% and (2) 33% fewer network links in the hemisphere ipsilateral to the stenotic vessel (Forero et al., 2017). The observation that atherosclerosis is associated with impaired NVC is further corroborated by Shroeter et. al (2006), who demonstrated that patients with cerebral microangiopathy (small vessel disease) had a weaker and delayed hemodynamic response compared with age-matched controls in both the left and right frontal lobes during a color-word Stroop task (Schroeter et al., 2007).

Given that our study includes only those with bilateral carotid plaques, it is unclear why the left hemisphere of the brain seems to be more affected than the right. This could be because the left side plays a more dominant role during the incongruous Stroop task (Jonides et al., 2000), thus making any deficits linked to vascular disease in that region more readily apparent. Measurements of plaque size and carotid intima medial thickness (cIMT) are currently ongoing, and will be used in future work to further assess the relationship between carotid atherosclerotic burden, plaque characteristics and cerebral hemodynamics.

Interestingly, the observed change in the pattern of brain activation was not accompanied by a change in central hemodynamic measures or task performance between people with and without atherosclerosis. Both groups found the incongruous Stroop task similarly ‘stressful’, and therefore may have had a similar level of sympathetic nervous system activation. There are several reasons that could explain the latter observation whereby the Plaque group maintained performance of the Stroop task despite experiencing reduced NVC in the regions assessed with fNIRS. One explanation could be the nature of the disease progression: hypoperfusion has been shown to *precede* cognitive decline, and must be chronic and persistent to cause the neuronal energy crisis that triggers neurodegeneration (Honjo, Black, Verhoeff, 2012, de la Torre, 2018). As the participants in the Plaque group had no prior evidence of CVD and the plaques were found incidentally (i.e. otherwise asymptomatic), we likely identified them at an early stage of the pathway between altered cerebral hemodynamics and measurable cognitive decline. It is also possible that regions outside of the areas interrogated by fNIRS in this study were recruited to maintain task performance in a phenomenon referred to as ‘neural compensation’ (Bondi, Houston, Eyler, Brown, 2005, Braskie, Small, Bookheimer, 2010, Wishart, 2006). Working under this supposition, cognitive impairment would then only be apparent when the additional cognitive reserve were no longer sufficient. Although plausible, this study does not provide the data support needed to determine whether neural compensation was occuring; this would require further work using a method capable of measuring global cerebral hemodynamics and neural activation. Finally, all participants of this study had an MMSE score of 24 or over; if this study were repeated on participants demonstrating signs of cognitive impairment, a relationship between hemodynamic measures and task performance might be observed.

**Fig. 7:**
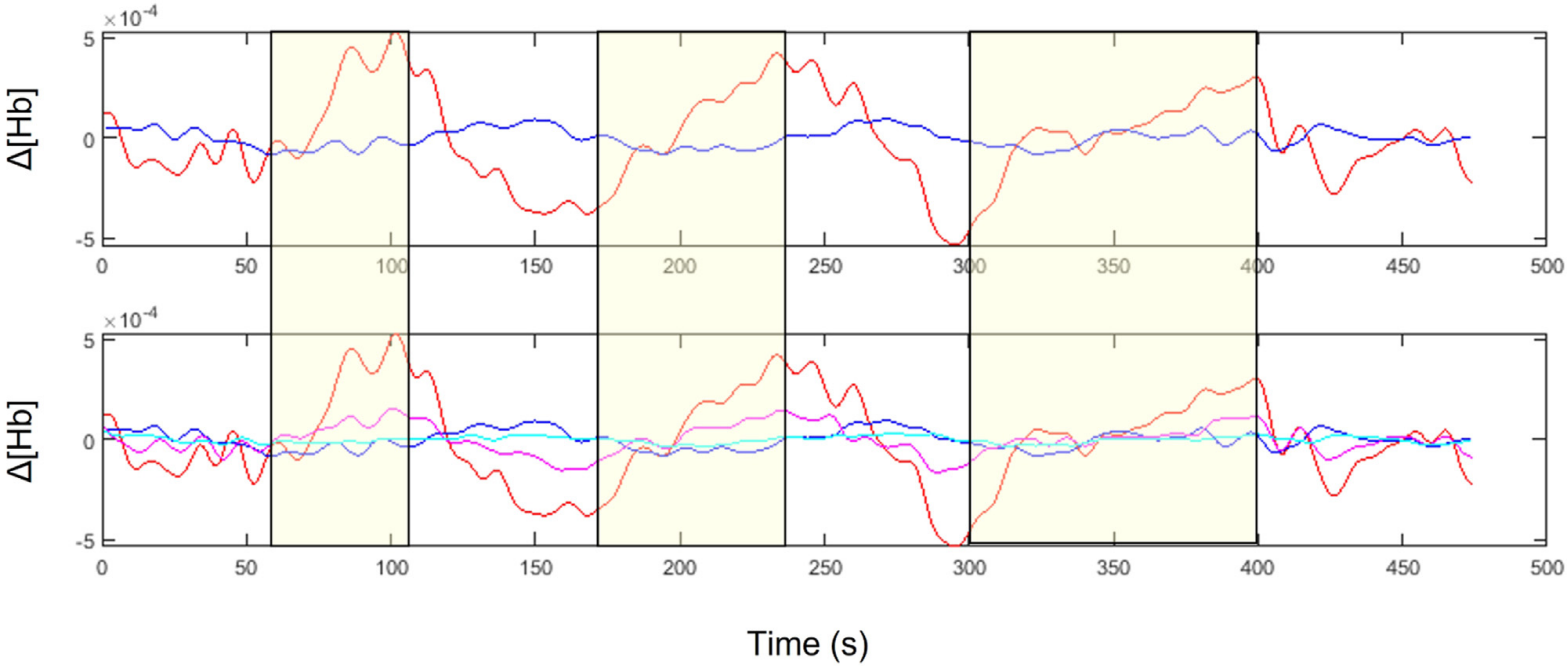
Top row: Example preprocessed channels from the left prefrontal cortex (LPFC) of a participant in the Healthy group with O2Hb and HHB shown in red and blue respectively. Note that in response to the stimulus periods (yellow blocks), the O2Hb signal increases to a greater extent corresponding decrease in HHB. Bottom row: The same channels as above are duplicated below, this time with the corresponding O2Hb (magenta) and HHB (cyan) measures from the short separation channel superimposed. Note that the O2Hb short separation channel responds in a similar way to the O2Hb long separation channel throughout the Stroop task, whereas the HHB short separation channel maintains a relatively flat shape. This implies that O2Hb is more sensitive to extracerebral contamination than HHB.

This study has several limitations. Firstly, the Stroop protocol was designed to be consistent with a previous INSIGHT wave before the addition of fNIRS. Consequently, the protocol was not modified to be optimal for fNIRS, which is why each condition in the Stroop test is administered in a single long block as opposed to multiple short blocks. Although every effort was made to minimize the effect of physiological contamination (such as including the short separation channels in the design matrix and specifying the contrast to compare the incongruous vs. the nominal condition), we cannot exclude that the cerebral component of the O2Hb fNIRS signal is confounded by extracerebral hemodynamics. Specifically, one concern is the spatial heterogeneity of scalp blood flow; we used a single short separation channel measurement to reflect the systemic effect for the entire hemisphere of the head, which is an imperfect method given the variability in systemic blood flow as a function of location. Although the HHB response is smaller in magnitude, it is less sensitive to this type of contamination (see Fig. 7) and thus is likely to be a better indicator of NVC in this studyTachtsidis and Scholkmann (2016). This is confirmed by our findings in our ‘known’ condition: we know that there should be increased activity in the PFC compared to the sensorimotor regions in response to the Stroop task. This was indeed picked up in the HHb signal, which showed a significantly greater decrease in HHb concentration on the LPFC than the RMC.However, we would like to emphasize that although O2Hb and HHB are not in perfect agreement, they are not necessarily contradictory. Rather, the increased concentration of O2Hb in response to the stimulus was more widespread whereas the decrease in HHB concentration was more localized. This pattern has been previously described in other fNIRS studies (Pinti et al., 2021).

Secondly, we chose to focus on the extremes of atherosclerosis within our sample (no plaques vs. bilateral plaques) to increase the chances of detecting differences with a limited sample size. In future it would be valuable to examine the relationship between NVC and atherosclerosis using atherosclerosis as a graded rather than a binary measure, although this is likely to require a larger sample size.

Although the Plaque and Healthy groups were age-matched and had relatively similar cardiovascular health profiles with the exception of the presence of carotid plaques, there was a difference in numbers of men between the two groups (70% men in the Plaque group versus 36% men in the Healthy group). We performed an additional LME analysis with statistical adjustment for sex (see supplementary information S2) and saw little effect on the overall results, but the sample size used was too small to draw any firm conclusions regarding the influence of sex on observed associations. Lastly, the sample was drawn from a UK-based national birth cohort and only included people of White European ethnicity and consequently our findings may not be generalizable to other samples.

This study also has many strengths which are worth highlighting. Firstly, we were able to take advantage of the advanced fNIRS preprocessing methods offered by SPM-fNIRS, such as customizing our design matrix to include physiological contaminants, and using pre-whitening to correct for serial autocorrelations in our data. Both of these have the effect of reducing the false positive rate, thereby giving us confidence that our results are robust even in the presence of noise. The Polhemus digitization and statistical parametric mapping also enabled us to preserve the geometry of our fNIRS measurements such that we could perform our analyses on anatomical regions rather than being restricted to channel-wise analyses. Secondly the extensive phenotyping performed as part of this study and previous data acquired as part of the NSHD enabled us to compare health and cognition status between our groups: this gives us some confidence that the differences observed in the cerebral hemodynamics between the two groups are likely to be a reflection of the differences related to carotid atherosclerosis.

## 5 Conclusions

As expected, evidence of brain activation based on fNIRS measurement of HHB was found in the LPFC of all participants. Despite individual heterogeneity of brain activation patterns, a consistent decrease in brain activity was observed in participants with bilateral carotid plaques compared with healthy controls during the incongruous Stroop task. Interestingly, this change in activation pattern was not accompanied by a marked differences in MAP, HR, or task performance. We demonstrate that carotid atherosclerosis, a well-established cardiovascular risk factor, may be associated with alterations in NVC while performing the Stroop task even in the absence of detectably impaired cognitive performance.

## Funding

Insight 46 is funded by grants from Alzheimer’s Research UK (ARUK-PG2014-1946, ARUK-PG2017-1946 PIs Schott, Fox, Richards), the Medical Research Council Dementias Platform UK (CSUB19166 PIs Schott, Fox, Richards), the Wolfson Foundation (PR/ylr/18575 PIs Fox, Schott), the Medical Research Council (MC_UU_12019/1 PI Kuh and MC_UU_12019/3 PI Richards), the Wellcome Trust (Clinical Research Fellowship 200109/Z/15/Z Parker) and Brain Research Trust (UCC14191, PI Schott). The cardiovascular assessments of Insight 46 are funded by a grant from the 10.13039/501100000274British Heart Foundation (PG17/90/33415 PI Hughes). Hughes also receives support from the 10.13039/501100000274British Heart Foundation, the Economic and Social Research Council (ESRC), the Horizon 2020 Framework Programme of the European Union, the National Institute on Aging, the National Institute for Health Research University College London Hospitals Biomedical Research Centre, the UK Medical Research Council.

## Disclosures

Nish Chaturvedi serves on a Data Safety and Monitoring Board for a clinical trial of a glucose lowering agent, funded by AstraZeneca. No other authors have competing interests.

## CRediT authorship contribution statement

**Sarah A. Mason:** Conceptualization, Writing – original draft, Software, Formal analysis, Visualization. **Lamia Al Saikhan:** Writing – review & editing, Investigation. **Siana Jones:** Writing – review & editing, Investigation. **Sarah-Naomi James:** Writing – review & editing, Data curation. **Heidi Murray-Smith:** Investigation, Project administration. **Alicja Rapala:** Project administration. **Suzanne Williams:** Investigation. **Carole Sudre:** Software, Writing – review & editing, Data curation. **Brian Wong:** Investigation. **Marcus Richards:** Writing – review & editing, Funding acquisition. **Nick C. Fox:** Writing – review & editing, Funding acquisition. **Rebecca Hardy:** Writing – review & editing, Funding acquisition. **Jonathan M. Schott:** Writing – review & editing, Funding acquisition. **Nish Chaturvedi:** Writing – review & editing, Funding acquisition. **Alun D. Hughes:** Conceptualization, Supervision, Formal analysis, Writing – review & editing, Funding acquisition.

## Appendix A Supplementary materials

Supplementary Data S1 Supplementary Raw Research Data. This is open data under the CC BY license http://creativecommons.org/licenses/by/4.0/ Supplementary Data S1

## References

[bib0001] Amieva H., Lafont S., Rouch-Leroyer I., Rainville C., Dartigues J.F., Orgogozo J.M., Fabrigoule C. (2004). Evidencing inhibitory deficits in Alzheimer’s disease through interference effects and shifting disabilities in the stroop test. Archives of Clinical Neuropsychology.

[bib0002] Bell R.D., Zlokovic B.V. (2009). Neurovascular mechanisms and blood-brain barrier disorder in Alzheimer’s disease. Acta Neuropathol..

[bib0003] Beratis I.N., Rabavilas A., Papadimitriou G.N., Papageorgiou C. (2010). Effect of handedness on the Stroop colour word task. Laterality.

[bib0004] Bondi M.W., Houston W.S., Eyler L.T., Brown G.G. (2005). Fmri evidence of compensatory mechanisms in older adults at genetic risk for alzheimer disease. Neurology.

[bib0005] Braskie M.N., Small G.W., Bookheimer S.Y. (2010). Vascular health risks and fmri activation during a memory task in older adults. Neurobiol. Aging.

[bib0006] Breteler M.M.B. (2000). Vascular risk factors for Alzheimer’s disease: an epidemiologic perspective. Neurobiol. Aging.

[bib0007] Bunce S.C., Izzetoglu M., Izzetoglu K., Onaral B., Pourrezaei K. (2006). Functional near-infrared spectroscopy. IEEE Eng. Med. Biol. Mag..

[bib0008] Cuevas K.U.o.C., CalkinsSusan D. (University of North Carolina G., Bell M.A.V.T. (2016). To stroop or not to stroop: sex-related differences in brain-behavior associations during early childhood. Psychophysiology.

[bib0009] Davidson D.J., Zacks R.T., Williams C.C. (2003). Stroop interference, practice, and aging. Aging, Neuropsychology, and Cognition.

[bib0010] De La Torre J.C. (2012). Cardiovascular risk factors promote brain hypoperfusion leading to cognitive decline and dementia. Cardiovasc Psychiatry Neurol.

[bib0011] Forero E., Novi S., Avelar W., Anjos C., Menko J., Forti R., Oliveira V., Cendes F., Covolan R., Mesquita R. (2017). Use of near-infrared spectroscopy to probe occlusion severity in patients diagnosed with carotid atherosclerotic disease. Medical Research Archives.

[bib0012] Friston K.J., Glaser D.E., Henson R.N., Kiebel S., Phillips C., Ashburner J. (2002). Classical and bayesian inference in neuroimaging: applications. Neuroimage.

[bib0013] Hardy R., Ghosh A.K., Deanfield J., Kuh D., Hughes A.D. (2016). Birthweight, childhood growth and left ventricular structure at age 60-64 years in a British birth cohort study. International Journal of Epidemiology.

[bib0014] Hofman A., Ott A., Breteler M.M., Bots M.L., Slooter A.J., Van Harskamp F., Van Duijn C.N., Van Broeckhoven C., Grobbee D.E. (1997). Atherosclerosis, apolipoprotein E, and prevalence of dementia and Alzheimer’s disease in the rotterdam study. Lancet.

[bib0015] Honig L.S., Kukull W., Mayeux R. (2005). Atherosclerosis and AD analysis of data from the US national Alzheimer’s. Neurology.

[bib0016] Honjo K., Black S.E., Verhoeff N.P.L.G. (2012). Alzheimer’s disease, cerebrovascular disease, and the β-amyloid Cascade. Canadian Journal of Neurological Sciences / Journal Canadien des Sciences Neurologiques.

[bib0017] Huppert T.J. (2016). Commentary on the statistical properties of noise and its implication on general linear models in functional near-infrared spectroscopy. Neurophotonics.

[bib0018] James S.N., Lane C.A., Parker T.D., Lu K., Collins J.D., Murray-Smith H., Byford M., Wong A., Keshavan A., Buchanan S., Keuss S.E., Kuh D., Fox N.C., Schott J.M., Richards M. (2018). Using a birth cohort to study brain health and preclinical dementia: recruitment and participation rates in insight 46. BMC Res Notes.

[bib0019] Jonides J., Marshuetz C., Smith E.E., Reuter-Lorenz P.A., Koeppe R.A., Hartley A. (2000). Age differences in behavior and PET activation reveal differences in interference resolution in verbal working memory. J Cogn Neurosci.

[bib0020] Jurcak V., Tsuzuki D., Dan I. (2007). 10/20, 10/10, and 10/5 systems revisited: their validity as relative head-surface-based positioning systems. Neuroimage.

[bib0021] Kearney-Schwartz A., Rossignol P., Bracard S., Felblinger J., Fay R., Boivin J.M., Lecompte T., Lacolley P., Benetos A., Zannad F. (2009). Vascular structure and function is correlated to cognitive performance and white matter hyperintensities in older hypertensive patients with subjective memory complaints. Stroke.

[bib0022] Khan A.A., Patel J., Desikan S., Chrencik M., Martinez-Delcid J., Caraballo B., Yokemick J., Gray V.L., Sorkin J.D., Cebral J., Sikdar S., Lal B.K. (2021). Asymptomatic carotid artery stenosis is associated with cerebral hypoperfusion. J. Vasc. Surg..

[bib0023] Kovacic J.C., Castellano J.M., Fuster V. (2012). The links between complex coronary disease, cerebrovascular disease, and degenerative brain disease. Ann. N. Y. Acad. Sci..

[bib0024] Kuh D., Pierce M., Adams J., Deanfield J., Ekelund U., Friberg P., Ghosh A.K., Harwood N., Hughes A., Macfarlane P.W., Mishra G., Pellerin D., Wong A., Stephen A.M., Richards M., Hardy R. (2011). Cohort profile: updating the cohort profile for the MRC national survey of health and development: a new clinic-based data collection for ageing research. Int J Epidemiol.

[bib0025] Kuh D., Wong A., Shah I., Moore A., Popham M., Curran P., Davis D., Sharma N., Richards M., Stafford M., Hardy R., Cooper R. (2016). The MRC national survey of health and development reaches age 70: maintaining participation at older ages in a birth cohort study. Eur. J. Epidemiol..

[bib0026] Laguë-Beauvais M., Brunet J., Gagnon L., Lesage F., Bherer L. (2013). A fNIRS investigation of switching and inhibition during the modified Stroop task in younger and older adults. Neuroimage.

[bib0027] Laird A.R., McMillan K.M., Lancaster J.L., Kochunov P., Turkeltaub P.E., Pardo J.V., Fox P.T. (2005). A comparison of label-based review and ALE meta-analysis in the stroop task. Hum Brain Mapp.

[bib0028] Lal B.K., Dux M.C., Sikdar S., Goldstein C., Khan A.A., Yokemick J., Zhao L. (2017). Asymptomatic carotid stenosis is associated with cognitive impairment. J. Vasc. Surg..

[bib0029] Lane C.A., Parker T.D., Cash D.M., Macpherson K., Donnachie E., Murray-Smith H., Barnes A., Barker S., Beasley D.G., Bras J., Brown D., Burgos N., Byford M., Jorge Cardoso M., Carvalho A., Collins J., De Vita E., Dickson J.C., Epie N., Espak M., Henley S.M., Hoskote C., Hutel M., Klimova J., Malone I.B., Markiewicz P., Melbourne A., Modat M., Schrag A., Shah S., Sharma N., Sudre C.H., Thomas D.L., Wong A., Zhang H., Hardy J., Zetterberg H., Ourselin S., Crutch S.J., Kuh D., Richards M., Fox N.C., Schott J.M. (2017). Study protocol: insight 46 - a neuroscience sub-study of the MRC National Survey of health and development. BMC Neurol.

[bib0030] Li C., Zheng J., Wang J., Gui L., Li C. (2009). An fmri stroop task study of prefrontal cortical function in normal aging, mild cognitive impairment, and alzheimers disease. Curr Alzheimer Res.

[bib0031] Li L., Cao D., Garber D.W., Kim H., Fukuchi K.I. (2003). Association of aortic atherosclerosis with cerebral β-Amyloidosis and learning deficits in a mouse model of Alzheimer’s disease. American Journal of Pathology.

[bib0032] Martin A. Lindquist, Ji Meng Loh, Lauren Y. Atlas, Tor D. Wage (2009). Modeling the hemodynamic response function in fMRI: efficiency, bias and mis-modeling. Neuroimage.

[bib0033] Mason S.A., Al Saikhan L., Jones S., Bale G., James S.-N., Murray-Smith H., Rapala A., Williams S., Wong B., Richards M., Fox N.C., Hardy R., Schott J.M., Chaturvedi N., Hughes A.D. (2020). Study protocol - Insight 46 cardiovascular: asub-study of the MRC national survey of health and development. Artery Res.

[bib0034] Mathiesen E.B., Waterloo K., Joakimsen O., Bonaa K.H. (2003). Neuropsychological test performance in asymptomatic carotid stenosis. Acta Neurol. Scand..

[bib0035] Milham M.P., Erickson K.I., Banich M.T., Kramer A.F., Webb A., Wszalek T., Cohen N.J. (2002). Attentional control in the aging brain: insights from an fMRI study of the stroop task. Brain Cogn.

[bib0036] Pinti P., Merla A., Aichelburg C., Lind F., Power S., Swingler E., Hamilton A., Gilbert S., Burgess P.W., Tachtsidis I. (2017). A novel GLM-based method for the automatic IDentification of functional events (AIDE) in fnirs data recorded in naturalistic environments. Neuroimage.

[bib0037] Pinti P., Siddiqui M.F., Levy A.D., Jones E.J., Tachtsidis I. (2021). An analysis framework for the integration of broadband NIRS and EEG to assess neurovascular and neurometabolic coupling. Sci Rep.

[bib0038] Prince M., Albanese E., Guerchet M., Prina M. (2014). Technical Report.

[bib0039] Purdon P.L., Weisskoff R.M. (1998). Effect of temporal autocorrelation due to physiological noise and stimulus paradigm on voxel-level false-positive rates in fmri. Hum Brain Mapp.

[bib0040] Rensink A.A., De Waal R.M., Kremer B., Verbeek M.M. (2003). Pathogenesis of cerebral amyloid angiopathy. Brain Res Rev.

[bib0041] Roberts K.L., Hall D.A. (2008). Examining a supramodal network for conflict processing: a systematic review and novel functional magnetic resonance imaging data for related visual and auditory stroop tasks. J Cogn Neurosci.

[bib0042] Romero J.R., Beiser A., Seshadri S., Benjamin E.J., Polak J.F., Vasan R.S., Au R., Decarli C., Wolf P.A. (2009). Carotid artery atherosclerosis, MRI indices of brain ischemia, aging, and cognitive impairment: the framingham study. Stroke.

[bib0043] Sabayan B., van Buchem M.A., Sigurdsson S., Zhang Q., Harris T.B., Gudnason V., Arai A.E., Launer L.J. (2015). Cardiac hemodynamics are linked with structural and functional features of brain aging: the age, gene/environment susceptibility (AGES)-Reykjavik study. J Am Heart Assoc.

[bib0044] Scarpina F., Tagini S. (2017). The stroop color and word test. Front Psychol.

[bib0045] Scholkmann F., Kleiser S., Metz A.J., Zimmermann R., Mata Pavia J., Wolf U., Wolf M. (2014). A review on continuous wave functional near-infrared spectroscopy and imaging instrumentation and methodology. Neuroimage.

[bib0046] Scholkmann F., Spichtig S., Muehlemann T., Wolf M. (2010). How to detect and reduce movement artifacts in near-infrared imaging using moving standard deviation and spline interpolation. Physiol Meas.

[bib0047] Scholkmann F., Wolf M. (2013). General equation for the differential pathlength factor of the frontal human head depending on wavelength and age. J Biomed Opt.

[bib0048] Schroeter M.L., Cutini S., Wahl M.M., Scheid R., Yves von Cramon D. (2007). Neurovascular coupling is impaired in cerebral microangiopathy-an event-related Stroop study. Neuroimage.

[bib0049] Schroeter M.L., Zysset S., Kruggel F., Von Cramon D.Y. (2003). Age dependency of the hemodynamic response as measured by functional near-infrared spectroscopy. Neuroimage.

[bib0050] Spieler D.H., Balota D.A., Faust M.E. (1996). Stroop performance in healthy younger and older adults and in individuals with dementia of the Alzheimer’s type. Journal of Experimental Psychology: Human Perception and Performance.

[bib0051] Stroop J.R. (1935). Studies of interference in serial verbal reactions. J Exp Psychol.

[bib0052] Tachtsidis I., Scholkmann F. (2016). Publisher’s note: false positives and false negatives in functional near-infrared spectroscopy: issues, challenges, and the way forward. Neurophotonics.

[bib0053] Tak S., Uga M., Flandin G., Dan I., Penny W.D. (2016). Sensor space group analysis for fnirs data. J. Neurosci. Methods.

[bib0054] de la Torre J. (2018). The vascular hypothesis of Alzheimer’s disease: akey to preclinical prediction of dementia using neuroimaging. J. Alzheimers Dis..

[bib0055] de la Torre J.C., Mussivand T. (1993). Can disturbed brain microcirculation cause Alzheimer’s disease?. Neurol Res.

[bib0056] Touboul P.J., Hennerici M.G., Meairs S., Adams H., Amarenco P., Bornstein N., Csiba L., Desvarieux M., Ebrahim S., Hernandez Hernandez R., Jaff M., Kownator S., Naqvi T., Prati P., Rundek T., Sitzer M., Schminke U., Tardif J.C., Taylor A., Vicaut E., Woo K.S. (2012). Mannheim carotid intima-media thickness and plaque consensus (2004-2006-2011). Cerebrovascular Diseases.

[bib0057] Udina C., Avtzi S., Durduran T., Holtzer R., Rosso A.L., Castellano-Tejedor C., Perez L.M., Soto-Bagaria L., Inzitari M. (2020). Functional near-infrared spectroscopy to study cerebral hemodynamics in older adults during cognitive and motor tasks: areview. Front Aging Neurosci.

[bib0058] Vanderhasselt M.A., de Raedt R., Baeken C. (2009). Dorsolateral prefrontal cortex and stroop performance: tackling the lateralization. Psychonomic Bulletin and Review.

[bib0059] Vendrell P., Junqué C., Pujol J., Jurado M.A., Molet J., Grafman J. (1995). The role of prefrontal regions in the Stroop task. Neuropsychologia.

[bib0060] Vernieri F., Silvestrini M., Tibuzzi F., Pasqualetti P., Altamura C., Passarelli F., Matteis M., Rossini P.M. (2006). Hemoglobin oxygen saturation as a marker of cerebral hemodynamics in carotid artery occlusion: an integrated transcranial doppler and near-infrared spectroscopy study. J. Neurol..

[bib0061] Wishart H. (2006). Increased brain activation during working memory in cognitively intact adults with the APOE ϵ4 allele. American Journal of Psychiatry.

[bib0062] Yücel M.A., Lühmann A.v., Scholkmann F., Gervain J., Dan I., Ayaz H., Boas D., Cooper R.J., Culver J., Elwell C.E., Eggebrecht A., Franceschini M.A., Grova C., Homae F., Lesage F., Obrig H., Tachtsidis I., Tak S., Tong Y., Torricelli A., Wabnitz H., Wolf M. (2021). Best practices for fNIRS publications. Neurophotonics.

[bib0063] Yücel M.A., Selb J., Aasted C.M., Petkov M.P., Becerra L., Borsook D., Boas D.A. (2015). Short separation regression improves statistical significance and better localizes the hemodynamic response obtained by near-infrared spectroscopy for tasks with differing autonomic responses. Neurophotonics.

[bib0064] Zysset S., Müller K., Lohmann G., Von Cramon D.Y. (2001). Color-word matching stroop task: separating interference and response conflict. Neuroimage.

