# Supplementary material for: Association between carotid atherosclerosis and brain activation patterns during the Stroop task in older adults: An fNIRS investigation
Source: Neuroimage. 2022 Aug 15;257:119302. doi: 10.1016/j.neuroimage.2022.119302 (PMC10466022; doi:10.1016/j.neuroimage.2022.119302)

**Supplementary Information**

S1: Linear mixed effects models of oxygenated hemoglobin (O2Hb) and deoxygenated hemoglobin (HHB) with Group (Healthy v Plaque) and Region (left prefrontal cortex, right prefrontal crotex, right motor cortex using the left motor cortex as the contrast) as fixed effects and including an interaction between Group x Region. Participant identifier was included as a random effect.


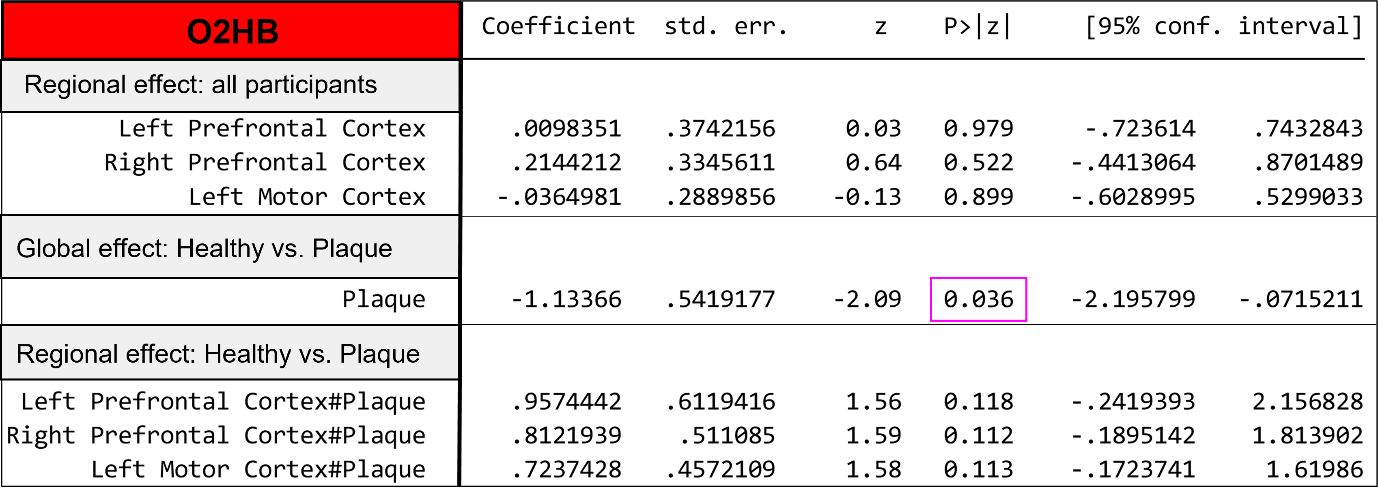


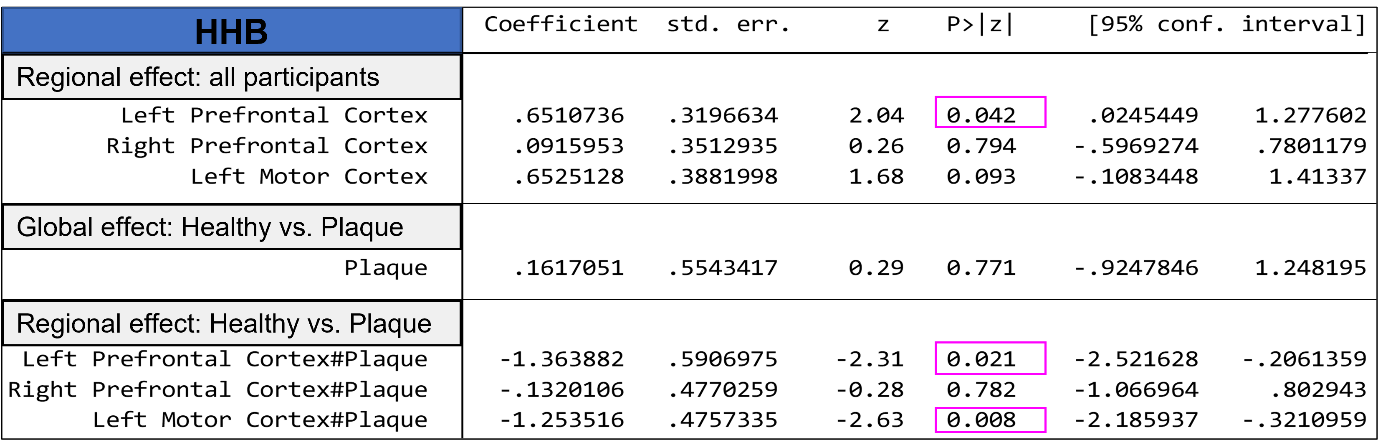


S2: Linear mixed effects model of deoxygenated hemoglobin (HHB) with Group (Healthy v Plaque) and Region (left prefrontal cortex, right prefrontal crotex, right motor cortex using the left motor cortex as the contrast) and sex as fixed effects and including an interaction between Group x Region. Participant identifier was included as a random effect.


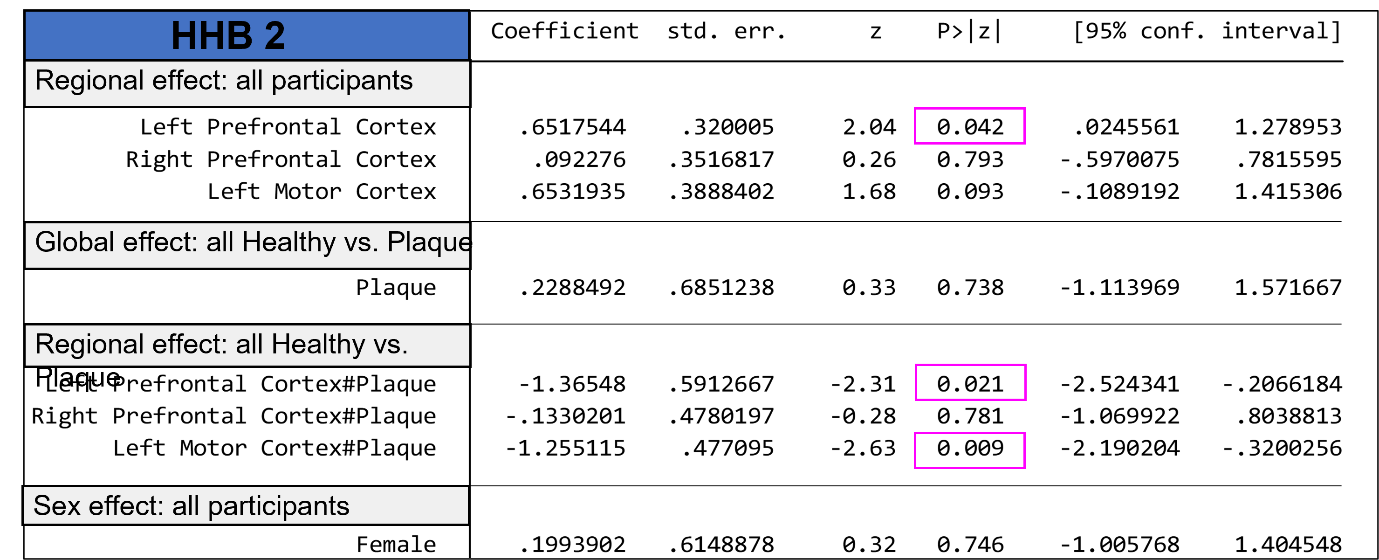

Supplement: Supplementary Data S1 — Supplementary Raw Research Data. This is open data under the CC BY license http://creativecommons.org/licenses/by/4.0/ [file mmc1.docx]
